# Effects of atrial natriuretic peptide on inter-organ crosstalk among the kidney, lung, and heart in a rat model of renal ischemia-reperfusion injury

**DOI:** 10.1186/s40635-014-0028-8

**Published:** 2014-11-08

**Authors:** Chieko Mitaka, May Khin Hnin Si, Miniwan Tulafu, Qi Yu, Tokujiro Uchida, Shinya Abe, Masanobu Kitagawa, Satoshi Ikeda, Yoshinobu Eishi, Makoto Tomita

**Affiliations:** Department of Critical Care Medicine, Tokyo Medical and Dental University Graduate School, 1-5-45, Yushima, Bunkyo-ku, Tokyo, 113-8519 Japan; Department of Anesthesiology, Tokyo Medical and Dental University Graduate School, 1-5-45, Yushima, Bunkyo-ku, Tokyo, 113-8519 Japan; Department of Comprehensive Pathology, Tokyo Medical and Dental University Graduate School, 1-5-45, Yushima, Bunkyo-ku, Tokyo, 113-8519 Japan; Department of Human Pathology, Tokyo Medical and Dental University Graduate School, 1-5-45, Yushima, Bunkyo-ku, Tokyo, 113-8519 Japan; Clinical Research Center, Tokyo Medical and Dental University Hospital of Medicine, 1-5-45, Yushima, Bunkyo-ku, Tokyo, 113-8519 Japan

**Keywords:** Acute kidney injury, Atrial natriuretic peptide, Cardiorenal syndrome, Cytokine, Lung inflammation, Ischemia-reperfusion, Rat

## Abstract

**Background:**

Renal ischemia-reperfusion injury (IRI) is a common cause of acute kidney injury and a frequent occurrence in critically ill patients. Renal IRI releases proinflammatory cytokines within the kidney that induce crosstalk between the kidney and other organ systems. Atrial natriuretic peptide (ANP) has anti-inflammatory as well as natriuretic effects and serves important functions as a regulator of blood pressure, fluid homeostasis, and inflammation. The objective of the present study was to elucidate whether ANP post-treatment attenuates kidney-lung-heart crosstalk in a rat model of renal IRI.

**Methods:**

In experiment I, a rat model of unilateral renal IRI with mechanical ventilation was prepared by clamping the left renal pedicle for 30 min. Five minutes after clamping, saline or ANP (0.2 μg/kg/min) was infused. The hemodynamics, arterial blood gases, and plasma concentrations of lactate and potassium were measured at baseline and at 1, 2, and 3 h after declamping. The mRNA expression and localization of tumor necrosis factor (TNF)-α, interleukin (IL)-1β, and IL-6 in the kidney, lung, and heart were examined. In experiment II, a rat model of bilateral renal IRI without mechanical ventilation was prepared by clamping bilateral renal pedicles for 30 min. Thirty minutes after clamping, lactated Ringer's (LR) solution or ANP (0.2 μg/kg/min) was infused. Plasma concentrations of TNF-α, IL-6, and IL-1β were determined at baseline and at 3 h after declamping.

**Results:**

In unilateral IRI rats with mechanical ventilation, ANP inhibited the following changes induced by IRI: metabolic acidosis; pulmonary edema; increases in lactate, creatinine, and potassium; and increases in the mRNA expression of TNF-α, IL-1β, and IL-6 in the kidney and lung and IL-1β and IL-6 in the heart. It also attenuated the histological localization of TNF-α, IL-6, and nuclear factor (NF)-κB in the kidney and lung. In bilateral IRI rats without mechanical ventilation, ANP attenuated the IRI-induced increases of the plasma concentrations of potassium, IL-1β, and IL-6.

**Conclusions:**

Renal IRI induced injury in remote organs including the lung and the contralateral kidney. ANP post-treatment ameliorated injuries in these organs by direct tissue protective effect and anti-inflammatory effects, which potentially inhibited inter-organ crosstalk.

## Background

Renal ischemia-reperfusion injury (IRI) is a common cause of acute kidney injury (AKI) in critically ill patients. Critically ill patients with AKI generally receive continuous renal replacement therapy, but the effects are insufficient to spare them from high mortality [1,2]. The high risk of death from AKI stems from extrarenal complications resulting from inter-organ crosstalk and multiple organ dysfunction syndrome [2,3]. Renal IRI exemplifies the pathophysiological significance of increased cytokine levels and enhanced inflammatory responses [4,5] that injure and inflame remote organs such as the lung [6] and heart [7].

Atrial natriuretic peptide (ANP) has natriuretic, diuretic, and vasodilating properties and serves important functions as a regulator of blood pressure and fluid volume homeostasis [8]. ANP increases the glomerular filtration rate (GFR) by dilating afferent arterioles and constricting efferent arterioles to increase the glomerular capillary hydraulic pressure [9]. It has also been found to enhance recovery from renal IRI by increasing the renal medullary blood flow in rats [10]. In a clinical setting, ANP infusion improves pulmonary capillary wedge pressure and cardiac index in patients with acute heart failure [11] and preserves renal function after cardiovascular surgery [12-14]. ANP has also been found to confer anti-inflammatory effects by inhibiting nuclear factor (NF)-κB activation and cytokine production [15-17]. In a recent study by our group, ANP pre-treatment prevented kidney-lung crosstalk in a rat model of renal IRI [18]. Yet it remains unclear whether ANP post-treatment protects the heart as well as lung after renal IRI. We hypothesized that the post-treatment might benefit the kidney, lung, and heart in a general fashion by attenuating inflammation. We divided the experiments into two parts, I and II. Our hypothesis of experiment I is that unilateral renal IRI induces inflammation on the contralateral kidney as well as remote organs and ANP post-treatment attenuates kidney-lung crosstalk by inhibiting expanding inflammation. Therefore, we examined the effects of IRI-induced inflammation on the contralateral kidney, lung, and heart in a rat model of unilateral renal IRI with mechanical ventilation and elucidated whether ANP post-treatment attenuates inter-organ crosstalk among the kidney, lung, and heart by inhibiting inflammation. Further, in experiment II, we adopted a rat model of bilateral renal IRI to bring our model somewhat closer to clinical reality. Our hypothesis of experiment II is that bilateral renal IRI induces kidney injury accompanied by increase in circulating cytokines and ANP post-treatment attenuates release of cytokines from the kidney into circulation. Therefore, we determined plasma cytokine concentration in the rat model of bilateral renal IRI excluding the effects of mechanical ventilation and saline and elucidated the inhibitory effect of ANP post-treatment on spreading inflammation.

## Methods

All the protocols in this study were approved by the Institutional Animal Care Committee of Tokyo Medical and Dental University (0140245A).

### Experiment I

#### Animal preparation

The animals were handled and cared for in accordance with the National Institutes of Health guidelines. Thirty-four male Sprague-Dawley rats (body weight 254 to 311 g) were anesthetized with an intraperitoneal injection of pentobarbital sodium (5 mg/100 g body weight). Each animal underwent a tracheostomy and intratracheal cannulation and was mechanically ventilated (Respirator Model SN-480-7, Shinano Ltd., Tokyo, Japan) under the following conditions: F_I_O_2_ 0.21, tidal volume of 10 ml/kg with 5 cmH_2_O positive end-expiratory pressure, respiratory rate of 30 to 40 cycles/min. The right carotid artery was cannulated with a catheter for continuous measurement of the arterial pressure and heart rate and for intermittent arterial blood sampling. The arterial pressure was measured with a blood pressure amplifier (AP-641G, SEN-6102M, Nihon Kohden, Tokyo, Japan) and data acquisition system (PowerLab2/26, ML826, ADInstruments, Bella Vista, Australia) by connecting the catheter to a transducer and calibrating at zero at the midchest. The right femoral vein was cannulated with a catheter for infusion of saline or ANP. The ANP was a generous gift from the Daiichi Sankyo Company (Tokyo, Japan).

#### Renal ischemia-reperfusion

The left renal pedicle was exposed via a midline incision, clamped with a vascular clip for 30 min, and released. Occlusion was verified visually by the change in the color of the kidney to a paler hue. After clamp removal, the restoration of the blood flow to the kidney was confirmed upon the return of the original color. The abdomen was closed in one layer. The sham surgery consisted of the same procedure, but with no clamping of the left renal pedicle. This renal ischemia-reperfusion injury is a model of AKI.

#### Experimental protocol

The rats were randomized to four experimental groups: an 1) IRI + saline group (*n* = 10), 2) IRI + ANP group (*n* = 10), 3) sham + saline group (*n* = 6), and 4) sham + ANP group (*n* = 8). All of the animals were mechanically ventilated. From 5 min after clamping of the left renal pedicle, the IRI + saline and sham + saline groups were infused with saline for 3 h 25 min at a rate of 6 ml/kg/h. The ANP infusion in the IRI + ANP and saline + ANP groups was started at the same time point (from 5 min after clamping of the left renal pedicle) and administered at the same rate and duration (0.2 μg/kg/min for 3 h 25 min) using saline mixed with ANP dissolved in 2-ml portions of distilled water. The heart rate, mean arterial pressure, arterial blood gases, and plasma concentrations of lactate, creatinine, and potassium were measured at baseline and at 1, 2, and 3 h after declamping. Blood gas analysis was performed on a blood gas analyzer (Radiometer ABL 837, Radiometer Medical ApS, Copenhagen, Denmark). At the completion of the experiment, all of the animals were killed with overdose of pentobarbital. The kidney, lung, and heart were harvested and either preserved at −80°C until use for the cytokine mRNA analysis or preserved in formalin until the histologic examination.

#### Wet/dry ratio of the lung

The wet/dry ratio of the lung is a gravimetric measure of pulmonary edema and an accurate gauge of changes in the lung dry mass [19]. We measured the wet/dry ratio by the same method reported by Heremans et al. [20] by desiccating the lung at 80°C until a constant weight was obtained. The ratio was calculated as a parameter of lung edema.

#### RNA extraction and TaqMan real-time PCR

Total RNA was extracted from the kidney, lung, and heart with TRIzol reagent (Invitrogen, Carlsbad, CA, USA) according to the manufacturer's instructions. The RNA concentration was determined by the absorbance read at 260 nm (GeneQuant 100, GE Healthcare UK Ltd, Chalfont St Giles, Buckinghamshire, UK). The primers and TaqMan probes for tumor necrosis factor (TNF)-α, interleukin (IL)-1β, IL-6, and glutaraldehyde-3-phosphate dehydrogenase (GAPDH) mRNA were purchased from a commercial laboratory (Applied Biosystems, Foster City, CA, USA). The mRNA expressions of TNF-α, IL-1β, and IL-6 were determined by real-time polymerase chain reaction (PCR). cDNA was synthesized using TaqMan reverse transcription reagents (Applied Biosystems, Roche Molecular Systems, Inc., Branchburg, NJ, USA) and quantified using a thermal cycler (PC707, ASTEC Co., Ltd., Minato-ku, Japan). TaqMan real-time PCR was performed using an ABI 7900HT (Applied Biosystems, Foster City, CA, USA). TaqMan rat GAPDH was used as an internal control and relative gene expression values were determined using the 2^−ΔΔCT^ method [21].

#### TNF-α, IL-6, and NF-κB immunostaining and scoring in the kidney, lung, and heart

Five rats from each group were used for the immunohistochemical examination. The kidney, lung, and heart were resected, embedded in paraffin, sliced into thin sections, and immunostained. Anti-TNF-α goat polyclonal antibody (SC-1348, diluted 1:20) and anti-IL-6 rabbit polyclonal antibody (SC-1265-R, diluted 1:200) were purchased from Santa Cruz Biotechnology (Dallas, TX, USA). Anti-NF-κB rabbit monoclonal antibody (1559-1, clone E381, diluted 1:200) was purchased from Epitomics (Burlingame, CA, USA). The sections were deparaffinized with xylene. For the IL-6 immunostaining, the sections were heat-treated in a microwave oven in citric acid buffer at pH 6.0 for 20 min and then air-cooled for 20 min. For the TNF-α and NF-κB immunostaining, the heat treatment was omitted. The subsequent immunostaining procedure was commenced by rehydrating the sections with an alcohol series and then treating them for 10 min with dH_2_O and H_2_O_2_ to inactivate the endogenous peroxidase. The antibodies were then added to the sections in a moisture chamber and reacted at RT for 3 h. After washing in phosphate buffer solution with Tween20 (PBST) for 30 min, the TNF-α samples were reacted for 30 min by indirect immunostaining using anti-goat antibody conjugated with horseradish peroxidase (P0449, diluted 1:30, DAKO, Tokyo, Japan). The IL-6 and NF-κB samples were visualized using a Novo Link Polymer Kit (RE7280-K, Leica Microsystems, Tokyo, Japan). After reacting the linker and polymer in the kit for 30 min each, the slides were visualized with diaminobenzidine, counterstained with hematoxylin, dehydrated, and cover-slipped. TNF-α, IL-6, and NF-κB expressions were evaluated semi-quantitatively by randomly choosing five areas in each slide and having them uniformly evaluated in a high-power field (×200) by a pathologist who had no knowledge of the experimental conditions (one of the authors). Scores of 3, 2, 1, and 0 were respectively assigned to fields with strong, moderate, weak, and negligible staining for each immunostaining. The level of expression was the mean value of five fields (TNF-α, IL-6, and NF-κB expression score, respectively).

### Experiment II

Thirteen male Sprague-Dawley rats (body weight 327 to 376 g) were anesthetized with an intraperitoneal injection of pentobarbital sodium (5 mg/100 g body weight). Each animal was allowed to breathe spontaneously, without mechanical ventilation. The right carotid artery was cannulated with a catheter and the arterial pressure was measured with a blood pressure amplifier as stated above. The right femoral vein was cannulated with a catheter for infusion of lactated Ringer's (LR) solution or ANP. The bilateral renal pedicles were clamped with vascular clips for 30 min and released. The rats were randomized to three groups: 1) IRI + LR group (*n* = 5), 2) IRI + ANP group (*n* = 5), and 3) sham + LR group (*n* = 3). From 30 min after clamping, the IRI + LR and sham + LR groups were infused with LR for 3 h at a rate of 6 ml/kg/h. The ANP infusion in the IRI + ANP group was started at the same time point and administered at the same rate and for the same duration (0.2 μg/kg/min for 3 h) using LR mixed with ANP dissolved in 2-ml portions of distilled water. The heart rate, mean arterial pressure, arterial blood gases, and plasma concentrations of lactate, creatinine, and potassium were measured at baseline and at 1, 2, and 3 h after declamping. The plasma concentrations of TNF-α, IL-1β, and IL-6 were determined at baseline and at 3 h using the rat ELISA kit (Quantikinetm TM, R&D Systems, Minneapolis, MN, USA) following the manufacturer's instruction. The samples were tested in duplicate.

### Statistical analysis

All data are shown as median and interquartile range (IQR). The hemodynamics; plasma concentrations of creatinine, lactate, potassium, and cytokines; and blood gas variables were all analyzed by the Kruskal-Wallis test at a fixed time point (3 h after declamping), as notable time-dependent changes in these parameters were found in repeated measures ANOVA for all three groups. The Kruskal-Wallis test was used to compare the wet/dry ratio, cytokine mRNA expression, and TNF-α, IL-6, and NF-κB scoring among the four groups. If the result from the Kruskal-Wallis test was significant, then the Mann-Whitney *U* test was similarly applied to analyze each pairing of groups. A *p* value of less than 0.05 was considered statistically significant.

## Results

### Experiment I

#### Changes in hemodynamic variables and plasma concentrations of creatinine and potassium

**Figure 1:**
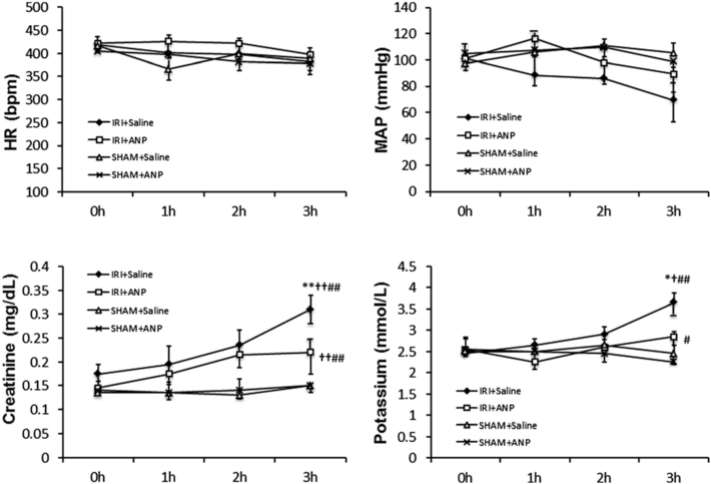
**Changes in the HR, MAP, and plasma concentrations of creatinine and potassium during mechanical ventilation.** Values are expressed as median. Vertical lines indicate the interquartile range (IQR). ANP, atrial natriuretic peptide; HR, heart rate; IRI, ischemia-reperfusion injury (unilateral); MAP, mean arterial pressure. *p < 0.05, **p < 0.01 vs. the IRI + ANP group; ^†^ *p* < 0.05, ^††^ *p* < 0.01 vs. the sham + ANP group; ^#^ *p* < 0.05, ^##^ *p* < 0.01 vs. the sham + saline group.

IRI did not induce any significant change in the heart rate or mean arterial pressure; however, IRI significantly (*p* < 0.05) increased the plasma concentrations of creatinine and potassium at 3 h. Post-IRI treatment by ANP prevented these changes in the variables related to renal function caused by IRI, and the values at 3 h in the IRI + ANP group were significantly lower than those in the IRI + saline group (Figure 1).

#### Changes in arterial blood gas variables, plasma lactate concentration, and lung wet/dry ratio

**Figure 2:**
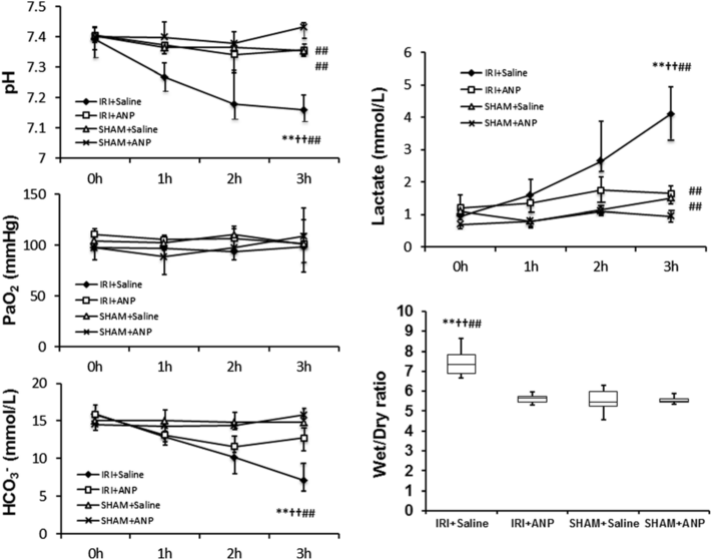
**Changes in arterial blood gas variables, lactate concentration, and lung wet/dry ratio during mechanical ventilation.** In blood gas variables and lactate concentration, value are expressed as median. Vertical lines indicate the interquartile range (IQR). In lung wet/dry ratio, boxes extend from the 25th to 75th percentile; the horizontal line shows the median. Error bars show the minimum and maximum. ANP, atrial natriuretic peptide; IRI, ischemia-reperfusion injury (unilateral). **p < 0.01 vs. the IRI + ANP group, ^††^ *p* < 0.01 vs. the sham + ANP group, ^##^ *p* < 0.01 vs. the sham + saline group.

IRI induced significant metabolic acidosis at 3 h (*p* < 0.01, Figure 2) with significantly elevated levels of plasma lactate (*p* < 0.05, Figure 2). Post-IRI treatment by ANP prevented IRI-induced metabolic acidosis and plasma lactate elevation. IRI significantly (*p* < 0.01) increased the lung wet/dry ratio, and ANP prevented this increase, as well (Figure 2).

#### Cytokine mRNA expression in the kidney, lung, and heart

**Figure 3:**
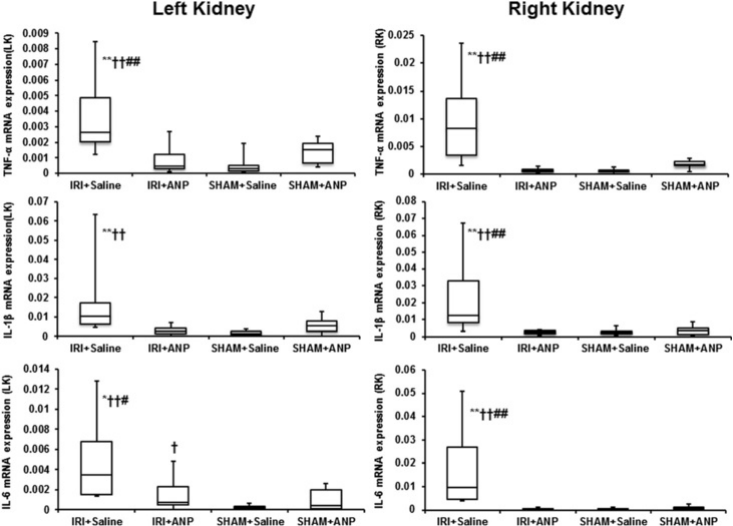
**Comparison of the mRNA expression of cytokines by TaqMan real-time PCR in the kidneys.** ANP, atrial natriuretic peptide; IL-1β, interleukin 1-β; IL-6, interleukin 6; IRI, ischemia-reperfusion injury; LK, left kidney; RK, right kidney; TNF-α, tumor necrosis factor-α. Boxes extend from the 25th to 75th percentile; the horizontal line shows the median. Error bars show the minimum and maximum. *p < 0.05, **p < 0.01 vs. the IRI + ANP group; ^††^ *p* < 0.01 vs. the sham + ANP group; ^#^ *p* < 0.05, ^##^ *p* < 0.01 vs. the sham + saline group.

**Figure 4:**
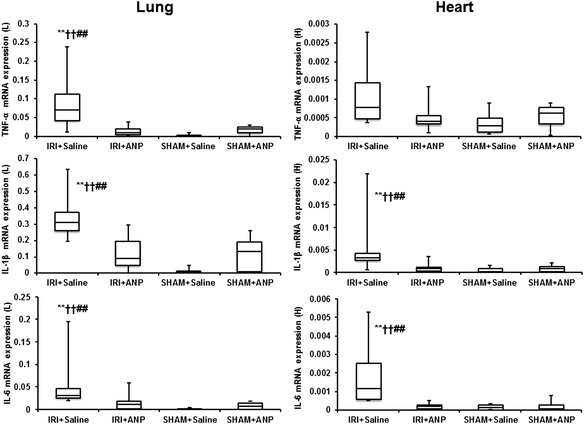
**Comparison of the mRNA expression of cytokines by TaqMan PCR in the lung and heart.** ANP, atrial natriuretic peptide; IL-1β, interleukin 1-β; IL-6, interleukin 6; IRI, ischemia-reperfusion injury; L, lung; H, heart; TNF-α, tumor necrosis factor-α. Boxes extend from the 25th to 75th percentile; the horizontal line shows the median. Error bars show the minimum and maximum. **p < 0.01 vs. the IRI + ANP group, ^††^ *p* < 0.01 vs. the sham + ANP group, ^##^ *p* < 0.01 vs. the sham + saline group.

Unilateral IRI significantly increased the mRNA expressions of TNF-α, IL-6, and IL-1β in both the ipsilateral kidney (*p* < 0.05 for IL-6; *p* < 0.01 for TNF-α and IL-1β) and the contralateral (right) kidney (*p* < 0.01 for TNF-α, IL-6, and IL-1β). Post-IRI treatment by ANP prevented the elevation in all these proinflammatory cytokines at 3 h after IRI (Figure 3). Furthermore, IRI significantly increased the mRNA expressions of TNF-α, IL-1β, and IL-6 in the lung (*p* < 0.01, Figure 4) and those of IL-1β and IL-6 in the heart (*p* < 0.01, Figure 4), and ANP prevented these elevations in the expression of the transcripts of proinflammatory cytokines in these remote organs (Figure 4).

#### Histological detection and localization of TNF-α, IL-6, and NF-κB in the kidney, lung, and heart

**Figure 5:**
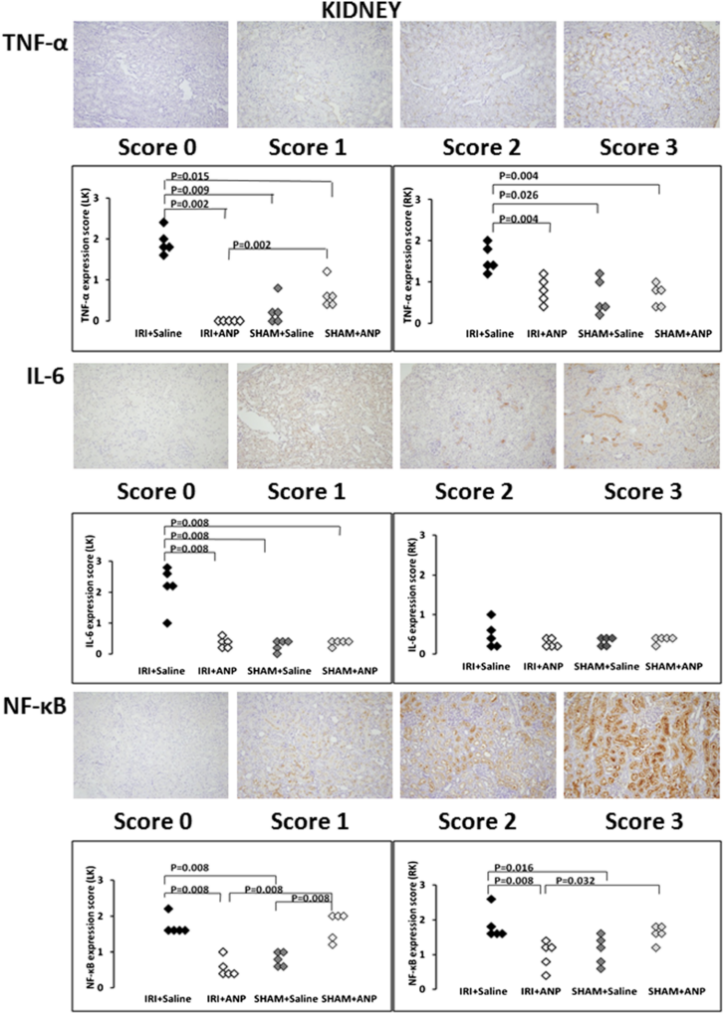
**Evaluation of TNF, IL-6, and NF-κB expressions in the kidney.** Upper figures: The evaluation of tumor necrosis factor (TNF)-α expression in the vascular endothelial cells of the kidney. TNF-α protein was stained in brown, and the level of TNF-α expression was scored: score 0 (hardly stained), score 1 (weakly stained), score 2 (moderately stained), score 3 (strongly stained). Comparison of the TNF-α expression scores in the left kidney (LK) and right kidney (RK). ANP, atrial natriuretic peptide; IRI, ischemia-reperfusion injury. Middle figures: The evaluation of interleukin (IL)-6 expression in the vascular endothelial cells and proximal convoluted tubules of the kidney. IL-6 protein was stained in brown, and the level of IL-6 expression was scored: score 0 (hardly stained), score 1 (weakly stained), score 2 (moderately stained), score 3 (strongly stained). Comparison of the IL-6 expression scores in the left kidney (LK) and right kidney (RK). Lower figures: The evaluation of nuclear factor (NF)-κB expression in the proximal convoluted tubules of the kidney. NF-κB protein was stained in brown, and the level of NF-κB expression was scored: score 0 (hardly stained), score 1 (weakly stained), score 2 (moderately stained), score 3 (strongly stained). Comparison of the NF-κB expression scores in the left kidney (LK) and right kidney (RK).

**Figure 6:**
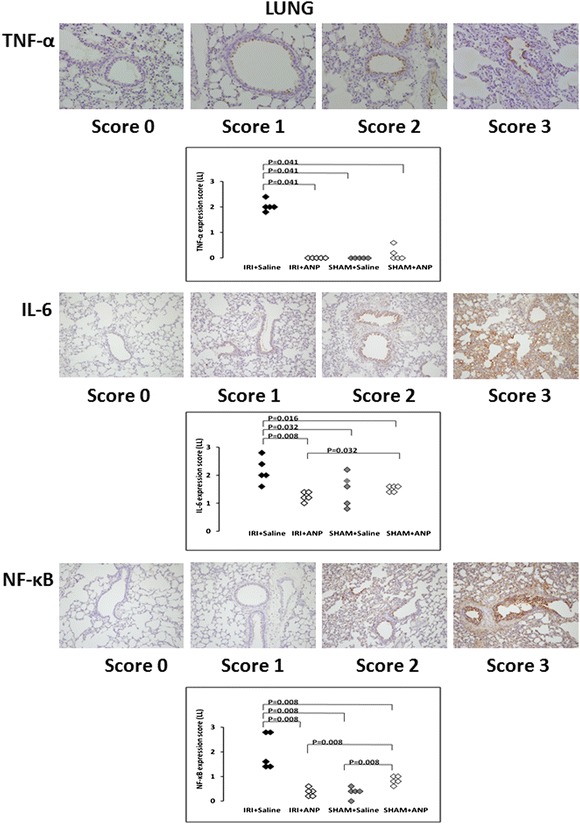
**Evaluation of TNF, IL-6, and NF-κB expressions in the lung.** Upper figures: The evaluation of tumor necrosis factor (TNF)-α expression in the bronchial epithelial cells of the lung. TNF-α protein was stained in brown, and the level of TNF-α expression was scored: score 0 (hardly stained), score 1 (weakly stained), score 2 (moderately stained), score 3 (strongly stained). Comparison of the TNF-α expression scores in the lung. ANP, atrial natriuretic peptide; IRI, ischemia-reperfusion injury. Middle figures: The evaluation of IL-6 in the columnar epithelial cells of the bronchioles of the lung. IL-6 protein was stained in brown, and the level of IL-6 expression was scored: score 0 (hardly stained), score 1 (weakly stained), score 2 (moderately stained), score 3 (strongly stained). Comparison of IL-6 expression score in the lung. Lower figures: The evaluation of nuclear factor (NF)-κB expression in the bronchioles of the lung. NF-κB protein was stained in brown, and the level of NF-κB expression was scored: score 0 (hardly stained), score 1 (weakly stained), score 2 (moderately stained), score 3 (strongly stained). Comparison of NF-κB expression score in the lung.

TNF-α was detected and localized in the vascular endothelial cells of the kidney, in the bronchial epithelial cells of the lung, and in the vascular endothelial cells of the heart. IL-6 was detected and localized in most vascular endothelial cells, in a few proximal convoluted tubules of the kidney, and in the columnar epithelial cells of the bronchioles of the lung and the vascular endothelial cells of the heart. NF-κB was detected and localized in the proximal convoluted tubules of the kidney, bronchioles of the lung, and myocardium of the heart. IRI significantly increased the TNF-α, IL-6, and NF-κB expression scores of the left (ipsilateral) kidney (*p* < 0.01) and the TNF-α and NF-κB expression scores of the right (contralateral) kidney (*p* < 0.01). Post-IRI ANP treatment prevented all these elevations (Figure 5). IRI significantly (*p* < 0.05) increased the TNF-α, IL-6, and NF-κB expression scores of the lung, and ANP prevented these increases (Figure 6), whereas IRI did not induce significant changes in the TNF-α, IL-6, and NF-κB expression scores in the heart.

### Experiment II

**Figure 7:**
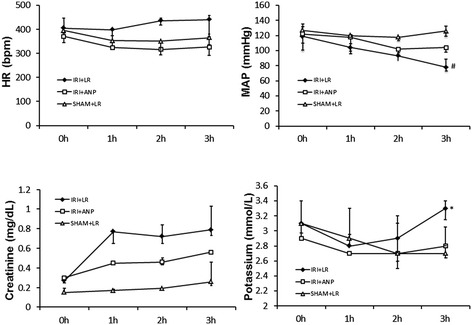
**Changes in the HR, MAP, and plasma concentrations of creatinine and potassium during spontaneous breathing.** Values are expressed as median. Vertical lines indicate the interquartile range (IQR). ANP, atrial natriuretic peptide; HR, heart rate; IRI, ischemia-reperfusion injury (bilateral); LR, lactated Ringer's solution; MAP, mean arterial pressure. *p < 0.05 vs. the IRI + ANP group, ^#^ *p* < 0.05 vs. the sham + LR group.

**Figure 8:**
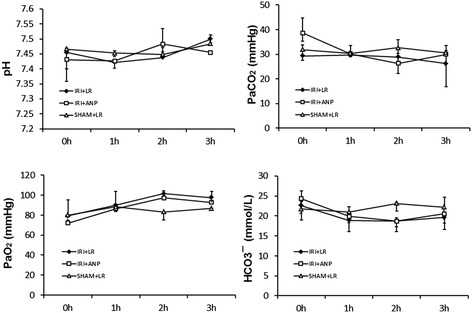
**Changes in arterial blood gas variables during spontaneous breathing.** Values are expressed as median. Vertical lines indicate the interquartile range (IQR). ANP, atrial natriuretic peptide; IRI, ischemia-reperfusion injury (bilateral).

**Figure 9:**
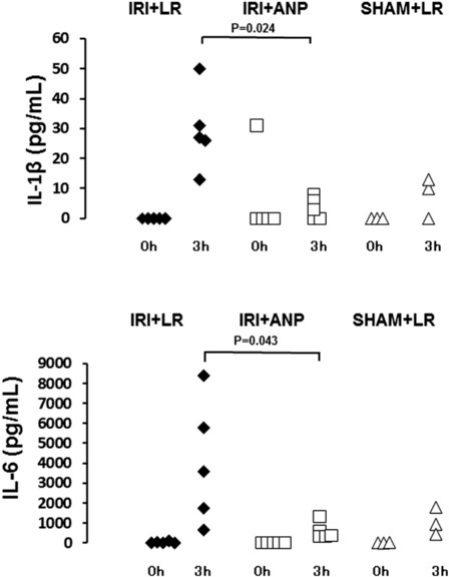
**Changes in plasma concentrations of IL-1β and IL-6 during spontaneous breathing.** ANP, atrial natriuretic peptide; IL-1β, interleukin 1-β; IL-6, interleukin 6; IRI, ischemia-reperfusion injury (bilateral); LR, lactated Ringer's solution.

Bilateral IRI procedures in the kidney did not change the heart rate, but significantly (*p* < 0.05) decreased the mean arterial pressure. IRI increased the plasma concentrations of creatinine and potassium, and ANP prevented the increase in the latter (Figure 7). IRI was not found to elicit acidosis by respiratory compensation and no significant change in arterial blood gas bicarbonate was observed (Figure 8). IRI also left the plasma lactate concentration unchanged. IRI significantly (*p* < 0.05) increased the plasma concentrations of IL-1β and IL-6, but not the concentration of TNF-α, and ANP attenuated the increases in IL-1β and IL-6 in 13 rats (Figure 9).

## Discussion

The most compelling findings observed in this rat model of unilateral renal IRI with mechanical ventilation (experiment I) were that unilateral renal IRI induced inflammation not only in the ipsilateral kidney but also in remote organs including the contralateral kidney, lung, and heart and ANP post-treatment inhibited inflammation of these organs. In addition, ANP post-treatment inhibited the renal IRI-induced metabolic acidosis, pulmonary edema, and increases in the plasma concentrations of lactate, creatinine, and potassium. Although unilateral renal IRI is not the main cause of AKI in critically ill patients, unilateral renal IRI remains a major problem in surgeries, such as renal transplantation [22] and juxtarenal and suprarenal abdominal aortic aneurysm repair [23]. The renal function of these patients must be preserved during the perioperative period. We therefore tried to investigate the effects of unilateral renal IRI on the non-ischemic contralateral kidney, as well as the lung and heart. Renal IRI augmented the mRNA expressions of TNF-α, IL-1β, and IL-6 in the kidney and lung, and this effect was inhibited by the ANP post-treatment. Renal IRI also augmented the mRNA expression of IL-1β and IL-6 in the heart, and the ANP post-treatment again inhibited the augmenting action. The ANP post-treatment prevented the renal IRI-induced localization of TNF-α, IL-6, and NF-κB in the kidney and IL-6 and NF-κB in the lung. Furthermore, in experiment II, the bilateral renal IRI increased the plasma concentrations of IL-1β and IL-6, but not the concentration of TNF-α, and the ANP treatment 30 min after clamping attenuated the increases in IL-1β and IL-6. The plasma TNF-α concentration might have passed its peak at 3 h. These results corroborate earlier evidence of an ANP-conferred enhancement of recovery from renal IRI in rats [10] and strengthen evidence favoring ANP as a possible treatment for AKI. In an earlier study by our group, ANP preserved renal function after suprarenal abdominal aortic cross-clamping in a dog model approximating ischemic AKI following abdominal aortic aneurysm repair [24]. In clinical studies, low-dose ANP infusion after cardiovascular surgery enhanced the renal excretory function, decreased the probability of dialysis, and improved the dialysis-free survival in ischemic acute renal failure [12-14]. The induction of IRI in the present study led to reduction in MAP in the saline group and LR group, but not in the sham group or ANP group. The plasma lactate concentration was also found to increase sharply in the IRI + saline group, but no such increase was observed after ANP treatment. These findings suggest that the administration of ANP might contribute to prevention of extravasation of the fluid, which resulted in maintenance of peripheral circulation. Our results showed successful results of ANP post-treatment in attenuating renal IRI and reducing cytokine mRNA expression in the kidney, lung, and heart. ANP post-treatment also reduced plasma cytokine (IL-1β and IL-6) concentrations, and this might be one of the mechanisms explaining the therapeutic effect of ANP on remote organ inflammation.

Renal IRI generally leads to outer medullary congestion and hypoxia, conditions that predispose patients to ischemic injury in the S_3_ segment of the proximal tubule [25]. ANP increases GFR by dilating the afferent arterioles and constricting the efferent arterioles to increase glomerular capillary hydraulic pressure [9]. The reno-protective effect of ANP may derive from protection against medullary ischemia via ANP-induced increases in the medullary vasa recta blood flow [10,26].

### Renal IRI and inflammation of remote organs

Renal IRI engages the innate and adaptive immune responses and works in conjunction with cytokine generation within the kidney [27]. Once this process starts, cellular and soluble mediators injure remote organs such as the lung and heart via organ crosstalk.

### Kidney-lung interaction

Renal IRI induced lung inflammation in a mouse model with systemic inflammatory syndrome and upregulated IL-6 mRNA expression in both the kidney and lung [6]. These findings are consistent with our present results. After renal IRI, we also detected and localized IL-6 in the columnar epithelial cells of the bronchioles of the lung and the vascular endothelial cells and proximal convoluted tubules of the kidney in our animals.

The alveolar epithelium has features in common with the renal tubular epithelium, such as localization of water channels and ion transporters [28]. Mechanisms of renal IRI-induced lung injury are assumed to include a dysregulation of water clearance, inflammation, an innate immune response, proinflammatory cytokines, oxidative stress, and apoptosis [29].

Studies confirming the expression of ANP and its receptors and their variable modes of regulation in the immune system [30] support the notion that ANP has immunomodulatory potency. ANP inhibits the activation of NF-κB production in both mouse macrophages and endothelial cells [15-17]. ANP post-treatment of our experimental animals inhibited the mRNA expression of TNF-α, IL-1β, and IL-6 in the kidney. The ability of ANP to suppress the induction of proinflammatory cytokines such as TNF-α, IL-1β, and IL-6 may signify a substantive anti-inflammatory action on the kidney and lung. ANP post-treatment was also found to inhibit the activation of NF-κB production in the kidney and lung in our study. These findings suggest that ANP has anti-inflammatory effects on both organs.

Increased capillary endothelial permeability is a major pathologic mechanism of pulmonary edema in acute lung injury and acute respiratory distress syndrome. Our group previously reported that ANP improved pulmonary gas exchange by reducing extravascular lung water in patients with acute lung injury [31] and in a canine model with oleic acid-induced pulmonary edema [32]. This finding is corroborated by reports that ANP knockout in mice increases the severity of lung inflammation and vascular barrier dysfunction caused by bacterial pathogens [33,34]. Increased ANP levels in patients with acute lung injury [35] may represent an important compensatory mechanism aimed at attenuation of injury and lung barrier dysfunction. Tian et al. [36] recently demonstrated a novel protective mechanism of ANP against pathologic hyper-permeability and suggested a pharmacological intervention for the prevention of increased vascular leak via PAK1-dependent modulation of guanine nucleotide exchange factor H1 activity. The activity of renal NF-κB appears to increase in the absence of the functional guanylyl cyclase/natriuretic peptide receptor-A (GC-A/NPRA) gene 1 and to elicit abnormalities by stimulating the synthesis of proinflammatory cytokines [37]. These findings, taken together, show that ANP protects the kidney by preventing proinflammatory cytokines via conterregulatory effects on NF-κB signaling.

### Kidney-heart interaction

Several inflammatory mediators participate in the pathophysiologic process of cardiorenal syndrome [38]. Increased production of inflammatory cytokines may adversely affect myocardial function. Elevated levels of circulating TNF-α and IL-6 are associated with the development of congestive heart failure and mortality in congestive heart failure patients [39,40]. Several different pathways, most notably the activation of inflammatory transcription factors and the induction of inflammatory genes and cytokines, may contribute to heart injury following renal IRI.

Renal IRI induced the mRNA expressions of TNF-α, IL-1β, and IL-6 in the heart, in our experiments, and ANP post-treatment attenuated the mRNA expressions of the latter two, IL-1β and IL-6. Yet the localizations of TNF-α, IL-6, and NF-κB in the heart at 3 h in the IRI + saline group were not significantly increased or significantly different from the localizations in other groups. These findings suggest that the cardiac levels of TNF-α, IL-6, and NF-κB were not increased in the heart 3 h after renal IRI. It may take more than 3 h to increase the cardiac levels of cytokines and NF-κB. In experiments with a rat model of renal IRI, Kelly [7] demonstrated an increase of circulating TNF-α by 1 h post renal ischemia, a further increase at 2 h, and steady elevation of the cytokine for 24 h. The cardiac levels of immnoreactive IL-1 and TNF-α in the same animals were elevated at 6, 24, and 48 h after renal ischemia, and echocardiography revealed left ventricular dysfunction, a likely sign of heart failure, at 48 h after renal IRI. It may take a longer time for renal IRI to induce hear failure.

### Inhibition of inter-organ crosstalk by ANP

It may be difficult to differentiate between inhibition of inter-organ crosstalk and direct organ protection, given that ANP has now been shown to confer protective effects on other organs in addition to the established anti-inflammatory effects. We know, however, that inter-organ crosstalk develops via cellular mediators such as neutrophils, macrophages, and lymphocytes, and inflammatory cytokines [29]. Matsumura et al. [41] have reported that ANP modulates the neutrophil functions and exerts protective effects against the neutrophil-induced endothelial cytotoxity. Chujo et al. [42] have also shown that ANP significantly inhibits IRI-induced increases in renal cytokine-induced neutrophil chemoattractant-1, a chemokine responsible for the activation of neutrophils and for neutrophil chemotaxis to sites of injury. Regarding plasma cytokine concentration which would be the main route of expansion of inflammation, ANP post-treatment attenuated IRI-induced elevation of the plasma concentrations of IL-1β and IL-6. We therefore suppose that ANP may both directly and indirectly disrupt the inter-organ crosstalk following renal IRI.

### Limitations of this study

There are some limitations to this study. First, in the present study, we have evaluated the effects of ANP on only renal IRI. Considering that there are multiple causes of AKI in critically ill patients (e.g., sepsis, nephrotoxic agents, hypoperfusion, and their combination) other than IRI, we cannot refer to the effects of ANP on AKI caused by other mechanisms. However, because it was notable that ANP post-treatment was effective to reduce tissue injury in the lung and kidney both in unilateral and in bilateral renal IRI, further study is needed to elucidate whether this beneficial effect might be observed in other pathophysiologic conditions. Second, saline infusion and mechanical ventilation might be an aggravating factor for organ injury in the present study. Unilateral renal IRI (experiment I) with mechanical ventilation induced significant metabolic acidosis. This result may have been due to a decrease in the renal blood flow by the positive pressure ventilation. Further, the applied tidal volume of 10 ml/kg might be a little too high to protect against lung injury. Bilateral renal IRI without mechanical ventilation (experiment II) did not induce acidosis by respiratory compensation, and the arterial blood gas bicarbonate was maintained by the infusion of LR. These findings suggest that the mechanical ventilation and saline both worsened the arterial blood gas parameters after the renal IRI. A recent clinical study has actually shown chloride-restrictive fluid infusion to be significantly associated with a significant decreased incidence of AKI and a significantly decreased use of renal replacement therapy in critically ill patients [43]. Therefore, we should consider the possibility that mechanical ventilation *per se*, ventilator setting, and type of infusion become exacerbation factors for organ dysfunction after the renal IRI. Nevertheless, it is noteworthy that ANP post-treatment has clearly prevented IRI-induced remote organ inflammation even in the condition with these kinds of aggravating factors.

## Conclusions

Unilateral renal IRI with mechanical ventilation induced inflammation not only in the ipsilateral kidney but also in remote organs including the contralateral kidney, lung, and heart. ANP post-treatment inhibited renal IRI-induced metabolic acidosis and the mRNA expression of TNF-α, IL-1β, and IL-6 in the kidney and lung and IL-1β and IL-6 in the heart. In addition, ANP post-treatment attenuated the IRI-induced increases in the plasma concentrations of IL-1β and IL-6, as well as the IRI-induced histological localization of TNF-α, IL-6, and NF-κB in the kidney and lung. These findings show that ANP conferred a reno-protective effect and anti-inflammatory effect both on the kidney and on the lung in the rat model of renal IRI. The cardiac levels of TNF-α, IL-6, and NF-κB were not significantly increased at 3 h after renal IRI, suggesting that renal IRI-induced heart injury may occur later than lung injury. Further studies are needed to elucidate the anti-inflammatory effects of ANP on the heart.

## Abbreviations

AKI: acute kidney injury
ANP: atrial natriuretic peptide
GFR: glomerular filtration rate
IL: interleukin
IRI: ischemia-reperfusion injury
NF-κB: nuclear factor-κB
TNF-α: tumor necrosis factor-α
